# Spatio-temporal variations and contextual factors of the supply of Airbnb in Rome. An initial investigation

**DOI:** 10.1007/s12076-022-00302-y

**Published:** 2022-05-17

**Authors:** Massimiliano Crisci, Federico Benassi, Hamidreza Rabiei-Dastjerdi, Gavin McArdle

**Affiliations:** 1 Italian National Research Council (CNR-IRPPS), Institute of Research on Population and Social Policies, Via Palestro 32, 00185 Rome, Italy; 2 Italian National Institute of Statistics, Via C. Balbo 16, 00144 Rome, Italy; 3 School of Computer Science and CeADAR, University College Dublin (UCD), Dublin, Ireland

**Keywords:** Airbnb, OLS model, Spatial regression models, Rome, South-Europe, C21, R12, R31

## Abstract

This paper offers an analysis of the supply of Airbnb accommodation in Rome, one of the main tourist destinations in the world, the third-largest city in Europe, by the number of Airbnb listings. The aim is to focus on the recent spatial trend of Airbnb listings, including the period of the COVID-19 pandemic, and highlight the main housing and socioeconomic characteristics of the neighbourhoods associated with a strong presence of Airbnb listings. The study is developed with quantitative methods and spatial regression (spatial lag and spatial error using OLS as a benchmark), based on data collected from the Inside Airbnb and Tomslee websites. In the period 2014–2019, the listing trend in Rome has been increasing in absolute numbers. After the start of the pandemic, the trend became negative, and the decline of Airbnb offerings is more substantial for shared accommodation. Airbnb supply is related to the distance from the city centre, the average income of the area, empty apartments, singles and the share of foreign residents coming from high-income countries. A signal of spatial diffusion of Airbnb listings emerges in the coastal area, even if they are increasingly concentrated in the historic centre, where there is a *monoculture* of short-term renting.

## Introduction

Airbnb is a peer-to-peer accommodation website and is a new driver of urban change globally (Carvalho et al. 2019; Guttentag 2019). Recently, a large body of literature has been produced about the Airbnb phenomenon, primarily by researchers from North America and Europe (Guttentag 2019) regarding different aspects of the short-term rental phenomenon, such as experience and motivations of guests and hosts, local impacts, tourism and regulations/policies (Nieuwland and Van Merlik 2020; Cheng and Jin 2019; Wachsmuth and Weisler 2018; Zervas et al. 2017; Guttentag 2015). In addition, findings from studies in Southern European urban contexts, where tourism has a central role in the local economy (Proença, Soukiazis 2008), have outlined local peculiarities and shown new processes of transnational gentrification linked to tourist flows from high-income countries and relatively low local housing costs that contributed to a displacement of lower-income residents from the city centres (see for instance: Garcia-López et al. 2019, López‐Gay et al. 2020 on Barcelona; Cocola-Gant and Gago 2019, Sequera and Nofre 2020 on Lisbon; Ardura Urquiaga et al. 2020 on Madrid; Jover and Díaz-Parra 2020 on Seville; Semi, Tonetta 2021 on Turin; Celata and Romano 2020, Gainsforth 2019 and Picascia et al. 2017 on other Italian urban areas). Research on the geographic dispersion of Airbnb rentals shows that listings are concentrated close to the main tourist attractions and facilities (Cocola Gant 2016; Gutiérrez et al. 2017; Benítez-Aurioles 2018; Guttentag 2019). Tourism drives a particular form of gentrification linked to the planetary rent gap, characterised by the inflow of a "mobile and floating population", largely consisting of young highly educated adults and foreign students, forcing the more sedentary residents to leave due to the increase of real estate values and worsening quality of life in neighbourhoods tailored to tourists (López‐Gay et al. 2020). Measures adopted by governments aimed to contain the spread of the COVID-19 pandemic severely impacted the activities of the tourism sector, including Airbnb, particularly in Italy and Spain, the first countries in Europe to be affected by the pandemic (Boros et al. 2020; Guglielminetti et al. 2021). Consequently, during 2020, Rome and Barcelona saw the most substantial decrease in the Airbnb reservation rate among the most highly internationalised megacities (Liang et al. 2021). Furthermore, research findings on the impacts of COVID-19 pandemic have shown that the interconnectedness of the world economy, through travel linkages as well as trade ties, can highlight spatial dependence and vulnerabilities (Krizstin et al. 2020; Rose et al. 2021).

In this article, a first investigation was developed using a case study in Rome, the third-largest city in Europe based on the number of Airbnb listings^1^, to highlight the main socioeconomic characteristics of the neighbourhoods where the supply of Airbnb accommodation is more diffused. In particular, the paper's objectives address the following research questions: (a) What is the evolution of Airbnb listings in the different areas of Rome from 2014 to 2020? (b) What are the neighbourhoods' housing, social, and economic characteristics where Airbnb listings are more present? (c) Do these characteristics change with the typology of the listings (entire apartment or private/shared room)?

The article is mainly based on data collected from the InsideAirbnb and Tomslee websites,^2^ which provides the opportunity to delineate the diffusion of short-term accommodation structures in many urban areas in space and time. Open access data are useful to detect how the activity of Airbnb is affecting neighbourhoods, for instance, producing gentrification and displacement of long-term residents and growth of housing prices (Wachsmuth and Weisler 2018). In this paper, Airbnb information has been related to socioeconomic and demographic contextual factors from different sources, following quantitative methods based on a spatial regression approach, through Spatial Lag Model (SAR) and Spatial Error Model (SEM) that are compared with traditional non-spatial OLS model. The work is structured as follows. In the next section, the study area, data and methodology are described. Then the results are presented and discussed. In the final section, some conclusions are proposed.

## Study area, data and methodology

This paper focuses on Rome, a south European metropolis, one of the main tourist destinations in the world, characterised by a high degree of heterogeneities in terms of social and economic structures (Barbieri et al. 2019), demographic profiles, and population groups (Clough Marinaro and Thomassen 2014). Over the last decades, the municipality of Rome has experienced an intense process of urban diffusion of the population from the urban core to the rest of the metropolitan area (Crisci, 2019). Nevertheless, the Italian capital is still monocentric and concentrates most relevant metropolitan functions in the inner city. Tourist accommodation is mainly situated in a specific portion of the urban core, the *historic centre*, located inside the ancient Aurelian walls, where the city's leading cultural and historical attractions are located.

The sources of Airbnb data we have used offer, *inter alia,* the point location of listings by typology (entire apartment, private and shared rooms) from August 2014.

Airbnb monthly data series of Rome show seasonality in the number of listings: the peak of the year is in August, followed by a fall and then a new rise starting from December (except for years 2019–20). Data completeness is a challenge, and for this reason, we have chosen to analyse the 2014–2020 time series considering only the peak month per year. Data for 2017 are limited to the first three months, so August was replaced with March as the closest dataset.

We analysed Airbnb listings regarding two categories: (1) entire apartments (2) private and shared rooms. According to the literature, short-term accommodation in "entire apartments" directly impacts the supply of real estate for long-term housing (Wachsmuth and Weisler 2018), while "private and shared rooms", in a certain way, could be considered part of the "shared nature" of Airbnb.

Other data concerning the study area were collected from various sources – Census, Population Registers, Italian National Revenue Agency – and processed to highlight different characteristics of the municipality of Rome at the neighbourhood scale (comprising of 155 urban zones or *zone urbanistiche*, from here on called: *Zurb*) according to four main dimensions: housing (5 variables), socio-demographic (6 variables), economic (1 variable) and geographic (1 variable) (Table1).^3^ These contextual variables have been widely used in other studies related to Italian urban contexts (see, for example, Barbieri et al. 2019) and represent well the different realities that characterise the relation of contemporary metropolitan areas with the Airbnb phenomenon (see for instance: Quattrone et al. 2016 on London; Dudás et al. 2017 and Jiao and Bai 2020 on New York and United States cities). The only data available to analyse the "housing" dimension are from the 2011 Italian census. Although these data are not recent, they show a situation that is most likely unchanged compared with today. In fact, in Rome, after the 2008 economic crisis, the real estate bubble burst, and, consequently, the building sector paused and is still struggling to recover (Coppola and Punziano 2018). The only pure geographical variable is represented by the "Euclidean Distance from the city's central area", which is a good *proxy* of the centrality or accessibility of each neighbourhood (Celata et al. 2020; Shabrina et al. 2021). The average distance from the city centre, Piazza Venezia^4^, for each Zurb polygon was calculated using Euclidean Distance and zonal statistics in ArcGIS. Euclidean Distance was used due to data limitation of public and transport networks and travel times.

**Table 1:** Contextual variables collected by 155 urban zones of Rome. Typology and source

Typology	Name	Description	Source
Housing	Empty (x_1)_	% empty dwellings	Census 2011
	Owned (x_2_)	% of families living in their own homes	Census 2011
	Rented (x_3_)	% of families in rented accommodation	Census 2011
	Detached (x_4_)	% detached or semi-detached houses (with 1–2 interiors)	Census 2011
	Large buildings (x_5_)	% flat in a large building (16 or more interiors)	Census 2011
Socio-demographic	Children (x_6_)	% 0–14 years old population	Population register 2019
	Young adults (x_7_)	% 20–34 years old population	Population register 2019
	Elderly (x_8_)	% population age 65 and over	Population register 2019
	Singles (x_9_)	% single-person households	Census 2011
	Ethnic LDCs (x_10_)	% foreigners from Less Advanced Countries on total residents	Population register 2019
	Ethnic MDCs (x_11_)	% foreigners from More Developed Countries on total residents	Population register 2019
Economic	Income (x_12_)	Deviation of income with respect to the average revenue of Rome's residents (average revenue = 1)	National Revenue Agency 2015
Geographical	Distance (x_13_)	Euclidean Distance from the central area of the city (raster transformation)	Our elaboration on Italian National Institute of Statistics data

a) MDCs stands for More Developed Countries. This group of countries includes: in Europe, the EU15 and EFTA countries; in the rest of the world, Australia, Canada, Israel, Japan, South Korea and the United States; b) LDCs stands for Less Developed Countries. This group comprises all the countries that are not classified as LDCs.

All 13 variables in Table 1 were used as covariates (i.e., contextual factors) (*xi*) for modelling two dependent variables that have been calculated as the percentage of whole apartments (variable y1) and private/shared rooms (variable y2) in Airbnb listings on the total real estate stock of each Zurb. The regression analysis was based on the comparison of three models: Ordinary Least Square (OLS), Spatial Lag Model (SAR) and Spatial Error Model (SEM). The first model is the classic linear standard regression model, and it is not a spatial model. SAR and SEM are spatial econometric models (LeSage and Pace 2009; Anselin 2001). Following Sun et al. (2020), a SAR model is a model that examines how the dependent variable (y) is influenced by the value assumed by the same variable in adjacent spatial units (in our case, Zurbs). The spatial lag parameter (ρ) refers to the estimate of how the average dependent variable in neighbouring spatial units (Zurbs) is associated with the same variable for a focal spatial unit (Zurb). By contrast, SEM estimates the extent to which the OLS residual of a Zurb is correlated with that in its adjacent Zurbs. The spatial error parameter (λ) measures the strength of the relationship between the average residuals/errors in neighbouring Zurbs and the residual/error of a given Zurb (Sun et al. 2020). In the analysis presented, all the spatial models and index (i.e., Moran's *I*) are based on a first-order Queen spatial weight matrix, which defines a neighbouring relationship between two Zurbs when they share a common boundary or vertex (Sun et al. 2020).

## Results

**Fig. 1:**
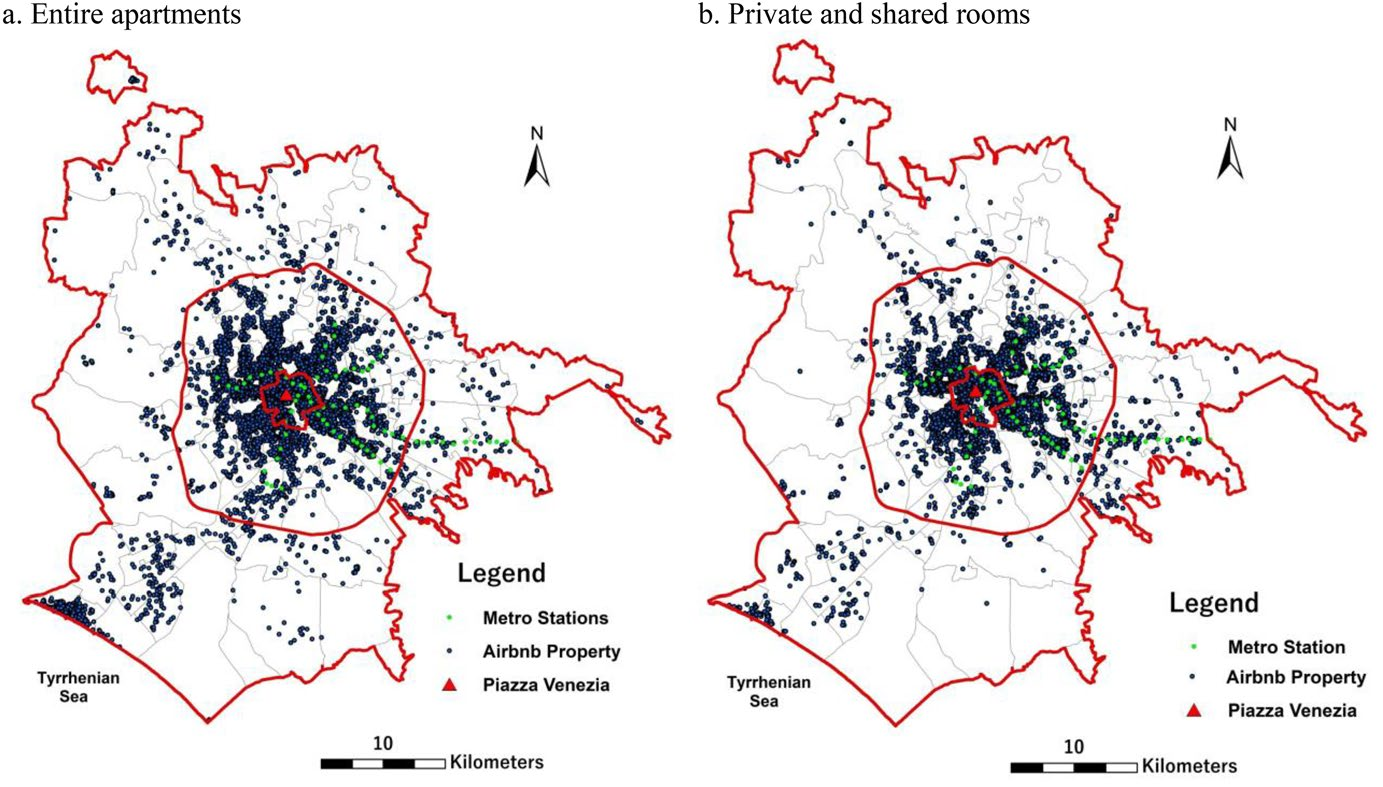
Distribution of Airbnb properties in the municipality of Rome. August 2020. *Note*: the maps show the metro stations, the location of the city centre (Piazza Venezia) and the spatial distribution of Airbnb properties in August 2020 in the 155 urban zones. In the maps, the borders of the three macro areas of Rome are highlighted with red lines, indicating: the municipality of Rome, the Great Ring Road, dividing the *Urban core* from the *Outer Periphery*; the ancient Aurelian Walls, surrounding the *Historic centre*. Source: Inside Airbnb website, http://insideairbnb.com/

The trend of Airbnb supply of entire apartments and private/shared rooms at a spatial level was analysed considering three macro areas (Fig. 1): the historic tourist centre, the urban core, and the outer periphery, outside the *Great Ring Road* surrounding Rome, which includes areas of urban sprawl and rural areas (the *agro romano*). Figure 1 provides a snapshot of the territorial distribution by typology of Airbnb listings in the municipality of Rome in August 2020. Entire apartments and private/shared rooms show a similar spatial allocation, markedly concentrated in the historic centre and neighbourhoods closer to the ancient city, particularly in the districts more accessible with public transportation. More peripheral districts, less dense neighbourhoods and those not connected with underground transport to the centre of the city have a smaller number of Airbnb properties, except for the South-western coastal district.

Generally, the Airbnb trend for the number of listings is increasing until August 2019, when the number of entire apartments reached over 20,000 units and private and shared rooms over 11,000 units (Table 2). If we consider that in August 2014, the total number of Airbnb listings in Rome was around 9,000, there has been an increase of more than three times in five years. The distribution of listings by the three macro areas shows similar absolute values for the historic centre and urban core. Still, the housing stock in the urban core's neighbourhoods is almost 14 times larger. Consequently, Airbnb listings on the total real estate stock are much higher in the historic centre than in the rest of the city. Considering only entire apartments, in August 2020, this percentage equals 12.5% in the historic centre, 1.0% in the urban core and 0.3% in the outer periphery. Private and shared rooms listings on total housing stock are 4.3% in the historic centre and less than one per cent in the rest of Rome. Thus, the Italian capital has a very high concentration of listings in the city centre. Similar results are seen in other Southern-European cities, such as Madrid (Urquiaga et al. 2020), Barcelona (López‐Gay et al. 2020) and Florence. Overall, two-thirds of the Airbnb listings in Rome are entire apartments (67.1% in August 2020). In the historic centre, entire apartment listings are much more present than shared accommodation (74% of total listings in 2020); in the urban core, the percentage of whole apartments is lower (64.6% in 2020), as empty houses are less numerous.

**Table 2:** Airbnb listings by typology and Rome's macro area from August 2014 to August 2020 *Source*: (a) from 2014 to 2016, Tom Slee website, http://tomslee.net; (b) from 2017 to 2020, Inside Airbnb website,

Macro area and listing typology	Airbnb listings (absolute values)							% of variation 2019–2020	% of listings on total real estate stock (August 2020
	Aug 2014	Aug 2015	Aug 2016	March 2017	Aug 2018	Aug 2019	Aug 2020		
*Entire apartments*									
Historic Centre	3212	5055	5918	6601	8275	9091	8436	−7.2	12.5
Urban Core	2808	5023	8179	8375	9695	10,258	9089	−11.4	1.0
Outer Periphery	NA	NA	519	631	986	1087	1023	−5.9	0.3
Rome (Total)	6022	10,080	14,616	15,607	18,956	20,436	18,548	−9.2	1.4
% Historic Centre	NA	NA	40.5	42.3	43.7	44.5	45.5		
*Private and shared rooms*									
Historic Centre	1040	1815	2456	2791	3110	3539	2908	−17.8	4.3
Urban Core	2102	3800	6237	6568	6832	7277	5635	−22.6	0.6
Outer Periphery	NA	NA	426	450	641	660	561	−15.0	0.2
Rome (Total)	3142	5618	9119	9809	10,583	11,476	9104	−20.7	0.7
% Historic Centre	NA	NA	26.9	28.5	29.4	30.8	31.9		

a) in the years 2014-2015, the number of listings in Rome is underestimated, due to the lack of data for many urban areas of the Urban Core and the whole area of the Outer Periphery; b) data of the year 2017 are incomplete, for this reason, the month of August is replaced with March; c) NA = not available.

In August 2020, the supply of Airbnb accommodation decreased with respect to the year before, as expected in a social distancing situation caused by COVID-19. This was also the effect of the dramatic decline in reservations in spring 2020, equal to over −40% with respect to the same period in 2019 (Liang et al. 2021). Such a decrease was more substantial for shared accommodation (−20.7%) than for entire apartments (−9.2%), confirming what emerged from other research: tourists were reluctant to book shared flats on Airbnb during the pandemic and preferred to rent complete properties (Bresciani et al. 2021). At the territorial level, the reduction of the Airbnb supply is more intense in the urban core, both for whole apartments (−11.4%) and for shared accommodation (−22.6%), than in the historic centre (respectively −7.2% and −17.8%). In the outer periphery, where Airbnb listings are less diffused, the COVID-19 related fall is more moderate. The amount of listings in the urban core and outer periphery is constantly rising. Still, in the historic centre, the increase is even faster and, as a consequence, in this area, the percentage of listings on the total of Rome is steadily growing in the period 2016–20, both for entire apartments (from 40.5 to 45.5%) and for shared accommodation (from 26.9 to 31.9%).

**Fig. 2:**
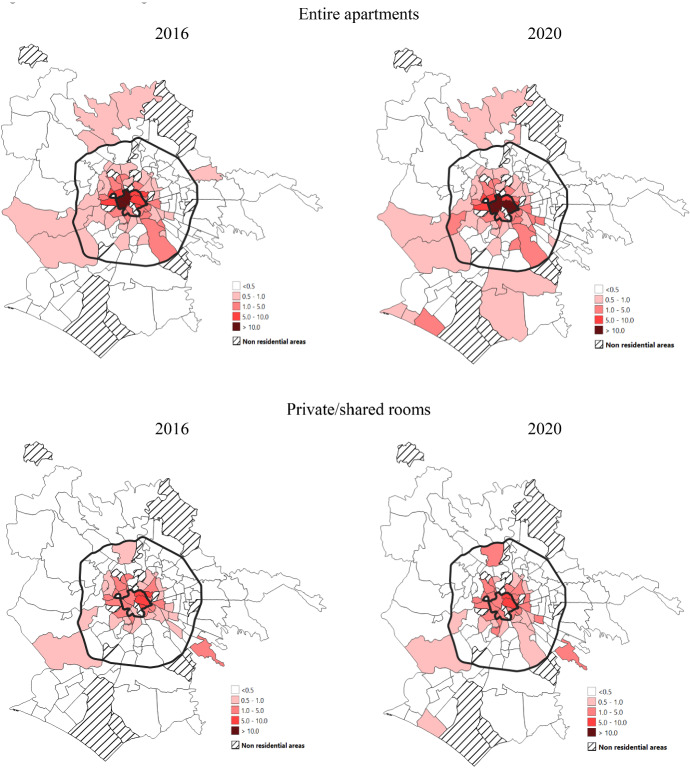
Percentage of entire apartments and private/shared rooms on the total real estate stock. August 2016 and August 2020. *Source*: our analysis on data from Inside Airbnb and Tom Slee websites and from Italian the National Institute of Statistics (ISTAT)

Spatial regression analysis applied here considers how the contextual variables of the 155 Zurbs affect the Airbnb offering in two points in time: August 2016, because of the lack of data in the years 2014 and 2015 in the urban core and outer periphery, and August 2020.^5^ A spatial approach has been used in such kinds of studies of Airbnb (Lagonigro et al. 2020; Gutiérrez et al. 2017). As previously described, our dependent study variables are represented by the percentage of entire apartments (y1) and private/shared rooms (y2) in Airbnb listings on total real estate stock by Zurb. Table 3 and Fig. 2 provides information about the spatiotemporal variability of these two variables. The level of global spatial autocorrelation (Moran's *I*) is relatively high for both variables and quite stable over time (Table 3): from 0.5 to 0.4 for y1 and 0.6 for y2 in 2016 and 2020. The positive sign of Moran's *I* implies positive spatial autocorrelation, i.e., similar values of y tend to cluster themselves on the base of a defined spatial structure of neighbourhoods. This evidence supports the need to use a spatial regression approach. From 2016 to 2020, the variability of y1 growth (standard deviation changes from 2.0 to 2.7 and CV from 2.2 to 2.3) while the one of y2 remains relatively stable (standard deviation is equal to 1.1 in both years and CV to 1.8).

**Table 3:** Statistical parameters and Moran's *I* index of spatial autocorrelation for entire apartments (y1) and private/shared rooms (y2) variables (i.e., % of listings on total real estate stock) in August 2016 and August 2020. *Source*: our analysis of data from Inside Airbnb and Tom Slee websites and the Italian National Institute of Statistics (ISTAT)

Statistical parameters	Entire apartments (y1)		Private and shared rooms (y2)	
	August 2016	August 2020	August 2016	August 2020
Min	0.0	0.0	0.0	0.0
Max	13.0	18.6	6.3	7.3
Q1	0.1	0.1	0.1	0.1
Median	0.2	0.4	0.2	0.2
Q3	0.7	0.8	0.5	0.5
IQR	0.6	0.7	0.5	0.5
Mean	0.9	1.2	0.6	0.6
Standard deviation	2.0	2.7	1.1	1.1
CV	2.2	2.3	1.8	1.8
Moran’s I (a)	0.5	0.4	0.6	0.6
N	155	155	155	155

p-value ≤ 0.05

From a spatial point of view (Fig. 2), the maps reveal that the higher percentages of the entire apartments (y1) are concentrated in the historic centre of Rome, where in 2020, 16.8% of the housing stock is for rent on the Airbnb website with even higher values in some specific sub-areas, such as the Zurbs Centro Storico (21.5%), Celio (19.3%) and Trastevere (19.1%). In 2020, we can also appreciate a sort of spatial diffusion process of Airbnb listings in the southern quadrant of the city. A growing Airbnb supply is emerging, especially along the coastline, where the neighbourhood of Ostia, an area that traditionally attracts Roman families, who move there in the summertime, is located. The Airbnb hosts of this area could attract groups of tourists coming from a broader range of places, potentially local, national and international. This general pattern of spatiotemporal variation occurs similarly for private and shared rooms (y2) but with a lower intensity.

To understand how and in which way the selected contextual variables (see Table 1) affect the dependent variables y1 and y2 in the Zurbs of Rome, we have controlled for spatial lag and spatial errors, obtaining valuable models, where problems of multicollinearity between variables have been handled. The variable selection for model specification was based on a complex strategy involving inserting and removing contextual variables from the original list to obtain a full significance OLS model with a high level of coherence in terms of net relationships between contextual and dependent variables.

At the end of this procedure, we have found the most effective models using five contextual variables broken down by Zurb^6^ (Table 4): percentage of empty dwellings (Empty, x_1_), percentage of single-person households (Singles, x_9_), percentage of foreigners from more developed countries (Ethnic MDCs, x_11_), average income (Income, x_12_) and Euclidean Distance from the city centre (Distance, x_13_). By using these five contextual variables, each of the four typologies of variables we considered (Housing, Socio-Demographic, Economic and Geographical) are represented. This reinforces the accuracy of the model's specification.

**Table 4:** Effect of contextual variables on the percentage of Airbnb listings on the total real estate values. Regression models (OLS, spatial lag and spatial error), August 2020^(a)^. *Source*: our elaboration on data from Inside Airbnb and Tom Slee websites and the Italian National Institute of Statistics (ISTAT).

Contextual variables	Entire apartments (y1)						Private and shared rooms (y2)					
	OLS		SEM		SAR		OLS		SEM		SAR	
	Estimate	SE	Estimate	SE	Estimate	SE	Estimate	SE	Estimate	SE	Estimate	SE
(Intercept)	0.6	1.28	2.68^**^	1.38	0.71	1.12	0.34	0.53	1.53^***^	0.6	0.41	0.41
Empty (x_1_)	0.05^*^	0.03	0.04	0.03	0.04	0.03	0.02^*^	0.13	0.01	0.01	0.02	0.01
Singles (x_9_)	0.05^**^	0.02	0.02	0.02	0.03	0.02	0.02^**^	0.01	−0.01	0.01	0.01	0.01
Ethnic MDCs (x_11_)	0.80^***^	0.19	0.37^**^	0.17	0.53^**^	0.16	0.16^**^	0.08	0.01	0.06	0.06	0.06
Income (x_12_)	−1.61^***^	0.61	−1.65^***^	0.59	−1.51^**^	0.53	−0.50^**^	0.25	−0.41^**^	0.02	−0.36^*^	0.19
Distance (x_13_)	−0.11^***^	0.04	−0.16^***^	0.06	−0.06^*^	0.04	−0.06^***^	0.02	−0.07^***^	0.06	−0.03^**^	0.04
λ (spatial error model)			0.63^***^						0.79^***^			
ρ (spatial lag parameter)					0.57^***^						0.73^***^	
AIC	695.93		676.82		670.591		423.952		367.763		368.358	
LL	−341.97		−332.41		−328.3		−205.976		−177.881		−177.179	
SC	714.19		695.08		691.9		442.212		386.024		389.662	

(a) For all the proposed models, multicollinearity between covariates is low since the multicollinearity condition number is always less than 30.

*p≤0.1, **p≤0.05, ***p≤0.01

Spatial regression models (SEM and SAR) outperform the non-spatial model (OLS) in terms of fit (see AIC, LL and SC parameters). These improvements are also confirmed by the high significance of the spatial lag and spatial error parameters. SAR is, in absolute, the model with the best fit, presenting lower values for each year of AIC and SC and higher values for each year of LL. However, when interpreting and comparing the estimation results, one must proceed with caution, given the particularity of the regression coefficients, especially in the case of the SAR model (Golgher and Voss, 2016). That is why we do not interpret the coefficients of the SAR model; instead, we compare the general performance of the models and the signs of the coefficients.

At first observation, OLS models produce essentially very similar results for apartments (y1) and rooms (y2), underlying the importance of the selected contextual variables in influencing the Airbnb dynamics in Rome. Empty (x_1_), Singles (x_9_), and Ethnic MDCs (x_11_) variables have a direct relationship with the dependent y1 and y2. In contrast, the variables Income (x_12_) and Distance (x_13_) are indirectly connected with the percentage of Airbnb listings on total real estate.

The introduction of the spatial dimension produces some changes and improvements. The spatial parameters (Lambda and Rho) result in positive and statistically significant regression results confirming that space matters. In terms of the covariate, what emerges is that for Airbnb listings of apartments (y1), three contextual variables are always statistically significant (OLS, SEM and SAR): Ethnic MDCs (direct relationship with y1), Income and Distance (indirect relationship with y1). It seems that Airbnb concerns less affluent areas. Regarding the percentage of private and shared rooms (y2), the only variables that are always statistically significant are Income and Distance.

In Table 5, we can appreciate the results of the same models (OLS, SEM and SAR) applied on 2020–2016 variation in the percentages of apartments (y1) and rooms (y2). Results show similarities and differences compared to those obtained previously (Table 4). In both cases, spatial models outperform the OLS model with a preference for the SAR model in the case of variation of y1 and for the SEM model in the case of variation of y2. Spatial parameters (Lambda and Rho) are always significant and positive concerning the y1 model and negative concerning y2. For the entire apartments, dependent variable (y1), the covariates that are statistically significant in all models are: Ethnic MDCs (x_11_) and Income (x_12_). The first contextual variable has a direct relationship with y1, while the other two have an inverse relationship with the dependent variable. These results are coherent with those in Table 4 for the entire apartments variable (y1) in 2020. The distance is statistically significant only in OLS model. On the other hand, all the covariates used in the model for the variation in the percentage of private and shared rooms (y2) results are not statistically significant. This variation depends on other contextual variables/factors not included in the model.

**Table 5:** OLS, SEM and SAR models for the variation of the dependent variable y1 and y2 in the period 2016–2020. *Source*: our analysis on data from Inside Airbnb and Tom Slee websites and from the Italian National Institute of Statistics (ISTAT)

Contextual variables	Entire apartments (y1)						Private and shared rooms (y2)					
	OLS		SEM		SAR		OLS		SEM		SAR	
	Estimate	SE	Estimate	SE	Estimate	SE	Estimate	SE	Estimate	SE	Estimate	SE
(Intercept)	−0.18	0.42	0.28	0.45	−0.06	0.39	−0.12	−0.18	−0.07	0.16	−0.11	0.18
Empty (x_1_)	0.01	0.01	0.01	0.01	0.01	0.01	0.01	0.01	0.01	0.01	0.01	0.01
Singles (x_9_)	0.01	0.01	0.01	0.01	0.01	0.01	−0.01	0.01	−0.01	0.01	−0.01	0.01
Ethnic MDCs (x_11_)	0.23^***^	0.06	0.15^***^	0.06	0.17^***^	0.06	0	0.03	0.01	0.02	0.01	0.02
Income (x_12_)	−0.39^**^	0.2	−0.47^**^	0.2	−0.41^**^	0.19	0.05	0.09	0.04	0.01	0.04	0.08
Distance (x_13_)	−0.01^*^	0.01	−0.02	0.02	−0.01	0.01	0.01	0.01	0.01	0.13	0.01	0.01
λ (spatial error model)			0.44^***^						−0.28^**^			
ρ (spatial lag parameter)					0.42^***^						−0.27^**^	
AIC	349.7		342.91		339.85		92.5		89.47		91.62	
LL	−168.85		−165.46		−162.93		−40.25		−38.73		−32.81	
SC	367.96		361.17		361.16		110.76		107.73		112.9	

(a) Multicollinearity between covariates is low for all the proposed models since the multicollinearity condition number is always less than 30.

*p≤0.1, **p≤0.05, ***p≤0.01

## Conclusions

This paper presents an analysis of the supply of Airbnb accommodation in the city of Rome, one of the main tourist destinations in the world, to identify the recent trend of Airbnb accommodation supply in different areas of the city, including the period of the COVID-19 outbreak, and to highlight the main housing and socioeconomic characteristics of the neighbourhoods associated with a strong presence of Airbnb listings according to the typology (apartments vs rooms).

To the best of our knowledge, this study represents one of the first attempts to analyse the territorial distribution of Airbnb listings in an Italian urban context through spatial regression methods. Since the Airbnb phenomenon is intrinsically a spatial process (e.g., Lagonigro et al. 2020), such an approach seems to produce less biased results than those obtainable with simple non-spatial linear regressions.

In 2014–2019, the listings trend was increasing in absolute numbers. Still, after the start of the pandemic and the first social distancing guidelines introduced in March 2020, the trend becomes negative, and the fall in Airbnb listings is stronger for shared accommodations (−20.7%) than for the entire apartments (−9.2%). A reduction of the Airbnb supply led to a dramatic decline in reservations in spring 2020, the highest fall of bookings among the major tourist cities in the world (Liang et al. 2021).

Not surprisingly, the tourist preference continues to be primarily concentrated in the historic centre, where the city's well-known attractions are located.

In the historic centre, the incidence of entire apartments on the Airbnb platform is much higher than in the urban core (74% vs 62% in 2020). This can be because the "shared nature" of Airbnb is more present in the peripheral neighbourhoods than in the historic part of the city, where the *monoculture* of the short-term rent rules, the residents renting a portion of their homes are fewer, and the apartments are often empty waiting for tourists (Cocola Gant 2016; Guttentag 2015). The incidence of Airbnb listings on total real estate stock is very high in the historic centre, considering only entire apartments 12.5% vs 1.0% in the urban core. The spatial regression models confirm that generally, the greater the distance from the city centre, the smaller is the Airbnb offering of entire apartments. Other interesting findings of the model are that Airbnb listings are less diffused in more affluent neighbourhoods, an outcome that deserves further investigation, and foreigners coming from richer countries can afford to live where the Airbnb phenomenon is more diffused conforming the role of Airbnb in fostering the "restructuring of social geography" of cities (López‐Gay et al. 2020).

Although Airbnb listings are not spread equally in the different areas of a city (Celata et al. 2020), in the case of Rome, there is evidence of a spatial diffusion process of the Airbnb supply in other peripheral areas, such as the coastal neighbourhood of Ostia. This could be a signal of a growing differentiation of the touristic offerings around Rome, not only based on the traditional historical-cultural heritage but also on the heterogeneity of its territory and on the "natural assets", combined with the fact that the seaside area is linked to Rome by train and is not far from Fiumicino International Airport. At the same time, many areas of the urban periphery near *The Great Ring Road* around Rome (the GRA) show a lesser number of Airbnb listings, probably due to the lack of public transport links and low natural quality.

At the end of 2020, Rome was the third-largest city in Europe regarding the number of Airbnb listings.^7^ We have seen that listings are concentrated in the central neighbourhoods of the city and tripled in the period 2014–2019. According to a large part of the literature (Wachsmuth and Weisler 2018; Guttentag 2019), it is expected that an increase in property values and a displacement in the long-term population of the inner city would have occurred. On the contrary, there was a decline in real estate values, and a process of re-urbanisation started, stopping, after four decades, the residential spread towards the hinterland (Crisci 2021). This phenomenon is undoubtedly linked to some specificities of the Roman context and will be interesting to highlight through a further in-depth study.

## Footnotes

1 According to the website Inside Airbnb (consulted the 18^th^ January of 2022), in Europe only London and Paris have more Airbnb listings than Rome.

2 Inside Airbnb < http://insideairbnb.com > ; Tom Slee < http://tomslee.net/ > .

3 Other variables from census 2011 on the economic dimension (such as, education, employment and unemployment) have been collected, but not considered in the elaboration of the models as biased.

4 Conventionally, the wide Piazza Venezia is considered the geographical city centre of Rome because it is very near to some of the most relevant places of the city – such as Colosseum, Capitol (Campidoglio) and Roman Forum—and the main streets of the historic city converge on it.

5 The 2014 and 2015 data from Tom Slee website don’t cover the entire municipality. For this reason, we have chosen to develop the models starting from 2016, as Tom Slee’s data become complete for the whole territory of Rome and consistent with the 2017–2020 data from Inside Airbnb website.

6 All the other results are available from the Authors on the basis of reasonable request.

7 See for instance < https://www.statista.com/statistics/815145/airbnb-listings-in-europe-by-city/ > .
